# Supplementary material for: Brain Atrophy Mediates the Relationship between Misfolded Proteins Deposition and Cognitive Impairment in Parkinson’s Disease
Source: J Pers Med. 2021 Jul 23;11(8):702. doi: 10.3390/jpm11080702 (PMC8401428; doi:10.3390/jpm11080702)
Supplement: Supplementary file 1 [file jpm-11-00702-s001.zip › jpm-1275258-SI.pdf]

Figure S1. ROC curve analysis of plasma misfolded proteins. Discrimination of PD and normal groups by p-Tau (pg/ml), Aβ42 (pg/ml), αSyn (fg/ml) and NfL (pg/ml).

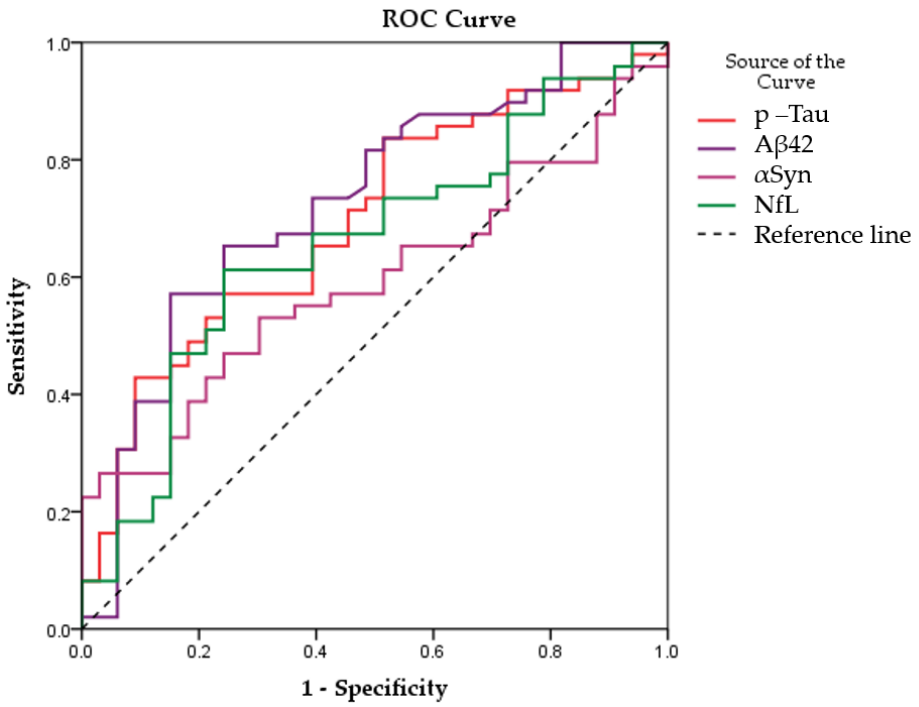

	**p-Tau**	**Aβ42**	**αSyn**	**NfL**
Cutoff-value	23.795	16.860	137.340	10.555
Sensitivity	0.429	0.571	0.265	0.612
Specificity	0.909	0.848	0.970	0.758
AUC	0.724	0.764	0.618	0.657
p	<0.001*	<0.001*	0.058	0.017*

Abbreviations: ROC curve (Receiver operating characteristic curve), p-Tau (phosphorylated-Tau), Aβ42 (amyloidβ-42), αSyn (α-synuclein), NfL (neurofilament light chain)

* Indicates p<0.05

We performed ROC curve analysis to evaluate plasma levels of p-Tau, Aβ42, αSyn and NfL in PD and normal groups. The cut-off value was chosen according to the maximum Youden's index (sensitivity + specificity-1). Statistical analysis was performed using SPSS version 20. The cut-off value for p-Tau is 23.795 pg/ml (sensitivity=42.9%, specificity= 90.9%, AUC= 0.724, p<0.001), 16.860 pg/ml for Aβ42 (sensitivity=57.1%, specificity= 84.8%, AUC= 0.764, p<0.001), 137.340 fg/ml for αSyn (sensitivity=26.5%, specificity= 97%, AUC= 0.618, p=0.058), and 10.555 pg/ml for NfL (sensitivity=61.2%, specificity= 75.8%, AUC= 0.657, p=0.017).
